# The role of cellular senescence in neurodegenerative diseases

**DOI:** 10.1007/s00204-024-03768-5

**Published:** 2024-05-15

**Authors:** Yating Wang, Kamil Kuca, Li You, Eugenie Nepovimova, Zbynek Heger, Marian Valko, Vojtech Adam, Qinghua Wu, Klaudia Jomova

**Affiliations:** 1 College of Life Science, Yangtze University, Jingzhou, 434025 China; 2 Department of Chemistry, Faculty of Science, University of Hradec Králové, 500 03 Hradec Králové, Czech Republic; 3 Biomedical Research Center, University Hospital Hradec Kralove, 500 05 Hradec Kralove, Czech Republic; 4 Andalusian Research Institute in Data Science and Computational Intelligence (DaSCI), University of Granada, Granada, Spain; 5 College of Physical Education and Health, Chongqing College of International Business and Economics, Chongqing, 401520 China; 6 Department of Chemistry and Biochemistry, Mendel University in Brno, 613 00 Brno, Czech Republic; 7 Faculty of Chemical and Food Technology, Slovak University of Technology, 812 37 Bratislava, Slovakia; 8 Department of Chemistry, Faculty of Natural Sciences, Constantine the Philosopher University in Nitra, 949 74 Nitra, Slovakia

**Keywords:** Cellular senescence, Neurodegenerative diseases, Alzheimer’s disease, Amyloid β · tau protein, Telomere shortening

## Abstract

Increasing evidence has revealed that cellular senescence drives NDs, including Alzheimer’s disease (AD) and Parkinson’s disease. Different senescent cell populations secrete senescence-associated secretory phenotypes (SASP), including matrix metalloproteinase-3, interleukin (IL)-1α, IL-6, and IL-8, which can harm adjacent microglia. Moreover, these cells possess high expression levels of senescence hallmarks (p16 and p21) and elevated senescence-associated β-galactosidase activity in in vitro and in vivo ND models. These senescence phenotypes contribute to the deposition of β-amyloid and tau-protein tangles. Selective clearance of senescent cells and SASP regulation by inhibiting p38/mitogen-activated protein kinase and nuclear factor kappa B signaling attenuate β-amyloid load and prevent tau-protein tangle deposition, thereby improving cognitive performance in AD mouse models. In addition, telomere shortening, a cellular senescence biomarker, is associated with increased ND risks. Telomere dysfunction causes cellular senescence, stimulating IL-6, tumor necrosis factor-α, and IL-1β secretions. The forced expression of telomerase activators prevents cellular senescence, yielding considerable neuroprotective effects. This review elucidates the mechanism of cellular senescence in ND pathogenesis, suggesting strategies to eliminate or restore senescent cells to a normal phenotype for treating such diseases.

## Introduction

The incidence of age-related neurodegenerative diseases (NDs), including Alzheimer’s disease (AD), Parkinson’s disease (PD), and amyotrophic lateral sclerosis (ALS), are increasing annually (Hou et al. 2019; Traxler et al. 2023). However, ND pathogenesis remains poorly understood. NDs are proteinopathies with misfolded intracellular protein aggregates (Gogia et al. 2023; Soto and Pritzkow 2018). Accumulated misfolded protein aggregates trigger detrimental processes, generating non-degradable oligomers and multimers, which induce cytotoxicity, cell death, and inflammation, leading to synaptic changes and neuronal cell loss (Tracy et al. 2022). AD, PD, and Huntington’s disease (HD) involve misfolded protein polymerization and deposition. For example, amyloid plaque neurofibrillary tangles (NFTs) in the brains of patients with AD and beta-amyloid (Aβ) and tau proteins in cerebrospinal fluid (CSF) indicate AD pathology in the brain (Gaikwad et al. 2021; Horie et al. 2020), Lewy bodies formed via α-synuclein aggregation in the PD brain (Fares et al. 2021), and huntingtin proteins in HD (Layburn et al. 2022). Autophagy is the primary intracellular mechanism underlying the degradation of accumulated misfolded proteins (Zhang et al. 2021). Defects in the autophagic pathway are associated with NDs at various stages; however, the exact autophagy regulation mechanism in the brain remains enigmatic (Basri et al. 2022). Furthermore, oxidative stress, metal-ion disorders, energy metabolism disorders, and immune inflammation contribute to ND pathogenesis (Rajesh and Kanneganti 2022; Zhang et al. 2023b). Nevertheless, the various manifestations are interrelated and complex. Moreover, no theory has accurately elucidated ND pathogenesis.

Increasing evidence shows cellular senescence involvement in ND pathogenesis (Buoso et al. 2022; Saez-Atienzar and Masliah 2020). Senescent cell accumulation accelerates organ aging and functional degradation and contributes to various NDs (Si et al. 2021; Skowronska-Krawczyk et al. 2015). The central nervous system (CNS) includes diverse cell types such as neurons, astrocytes, microglia, neural stem cells, and oligodendrocytes (Bigbee 2023). Senescence in these cell types is related to several NDs, including AD, PD, ALS, and Down syndrome (Ng et al. 2023; Preininger and Kaufer 2022). Cellular senescence may be a fundamental mechanism underlying many complicated cellular responses during neurodegenerative lesion progression (Sahu et al. 2022). In colonic tissues of AD mice, the levels of two cellular senescence markers, integrin β3 and senescence-associated β-galactosidase (SA-β-gal), increased with age (Tun et al. 2023). Cellular senescence can disrupt proteomic function and homeostasis, altering the normal rates of protein synthesis and degradation and producing misfolded proteins or abnormal protein aggregates in AD, PD, and HD (Martinez-Cue and Rueda 2020). Moreover, the accumulation of double-stranded deoxyribonucleic acid (DNA) breaks and cellular senescence are intermediaries in the pathogenesis of α-synuclein-induced PD (Yoon et al. 2022). Cellular senescence markers appear earlier in the brain tissue of AD mice than neuronal loss or AD-like cognitive impairment onset. Therefore, a potential accumulation of pathogenic senescent cells has been observed during this disease progression (Dorigatti et al. 2022). Targeted depletion of senescent cells in spinal-cord-injured mice improves motor, sensory, and bladder function, which is related to improved myelin sparing, reduced inflammation, and decreased secretion of pro-inflammatory factors (Paramos-de-Carvalho et al. 2021). As a driver of NDs, cellular senescence may initiate a positive feedback loop that accelerates neurodegeneration progression. The characteristics and inducements of cellular senescence are similar to those of neuropathological events, signifying its pivotal role in ND pathogenesis (Lu et al. 2023).

In this review, we aim to comprehensively outline the recent research advances in elucidating molecular mechanisms underlying the role of cellular senescence in ND pathogenesis. We discussed the brain cell senescence in neurodegeneration and its role in Aβ accumulation, tau-mediated disease, and telomere damage, which mediate NDs. We further discussed diagnosis and therapy strategies targeting senescence and NDs**.** Finally, we outlined the major challenges for future experiments in this context. Eliminating senescent cells might be an important target for treating NDs.

## Brain-cell senescence contributes to neurodegeneration

Cells in brain tissue play different functional roles and can influence and interact with each other (Chien et al. 2023). Numerous studies reveal senescence-like traits and markers in various nervous system cell populations in in vitro and in vivo models of NDs (Moiseeva et al. 2023; Russo and Riessland 2022). Increased astrocytic senescence was observed in postmortem PD brain samples (Simmnacher et al. 2020). Senescent astrocytes may be involved in sporadic PD development, as demonstrated by alleviated neurodegeneration in a PD mouse model upon senescent cell elimination (Chinta et al. 2018). In the ALS rodent model, the ability of astrocytes to support motoneurons decreases with senescence (Das and Svendsen 2015). Moreover, senescent astrocytes lead to neurodegeneration, including AD and related dementias (Bhat et al. 2012; Limbad et al. 2020). Senescent astrocytes exhibit increased secretion of senescence-associated secretory phenotype (SASP) factors, impaired physiological function, and dysfunctional mitochondria, producing elevated levels of reactive oxygen species (ROS). These factors activate the nuclear factor-κB (NF-κB) pathway and stimulate interleukin (IL)-6 and interferon-γ secretions, subsequently triggering Aβ accumulation, tau hyperphosphorylation, and NFT deposition in AD (Gao et al. 2021; Han et al. 2020). In addition, senescent astrocytes increase glutamate release, inducing oligodendrocyte senescence (Limbad et al. 2020).

Oligodendrocytes are produced by adult oligodendrocyte progenitor cells (OPCs), which are the sole source of myelination in the CNS (Spitzer et al. 2019). The senescence of progenitor cells is one of the reasons for the decrease of remyelination potential in patients with progressive multiple sclerosis (Nicaise et al. 2019). In addition to AD neuronal pathology characteristics, white matter abnormalities, particularly those involving myelin and oligodendrocytes, have important implications in AD pathogenesis (Nasrabady et al. 2018). Myelin disruption is associated with cognitive decline in patients with AD. Oligodendrocytes play a supporting and regulating role in neurons, and the myelin sheath is damaged under hypoxia, leading to decreased neurons (Wang et al. 2018). OPC senescence, including tissue stiffness, is the earliest neuropathological change in the brain (Segel et al. 2019). In a transgenic AD mouse model, OPC senescence in the hippocampus was an early AD marker accelerating myelin loss and cognitive decline (Vanzulli et al. 2020). Rodent and human OPCs display heightened sensitivity to oxidative stress, which is related to PD, as this disease is characterized by oxidative stress and pathogenic gene variants associated with mitochondrial damage (Akay et al. 2021; Boda et al. 2022; De Nuccio et al. 2020). In addition, cellular senescence contributes to the pathogenesis (Si et al. 2021). In the brain tissue of patients with PD, α-synuclein deposition correlates with increased senescent cell accumulation and higher SA-β-gal expression (Bae et al. 2022). The senescent cell marker p16 and several SASP factors, including MMP-3, IL-1α, IL-6, and IL-8, are increased in PD brain tissues, indicating that cellular senescence causes dopaminergic neurodegeneration (Chinta et al. 2018; Si et al. 2021).

**Fig. 1:**
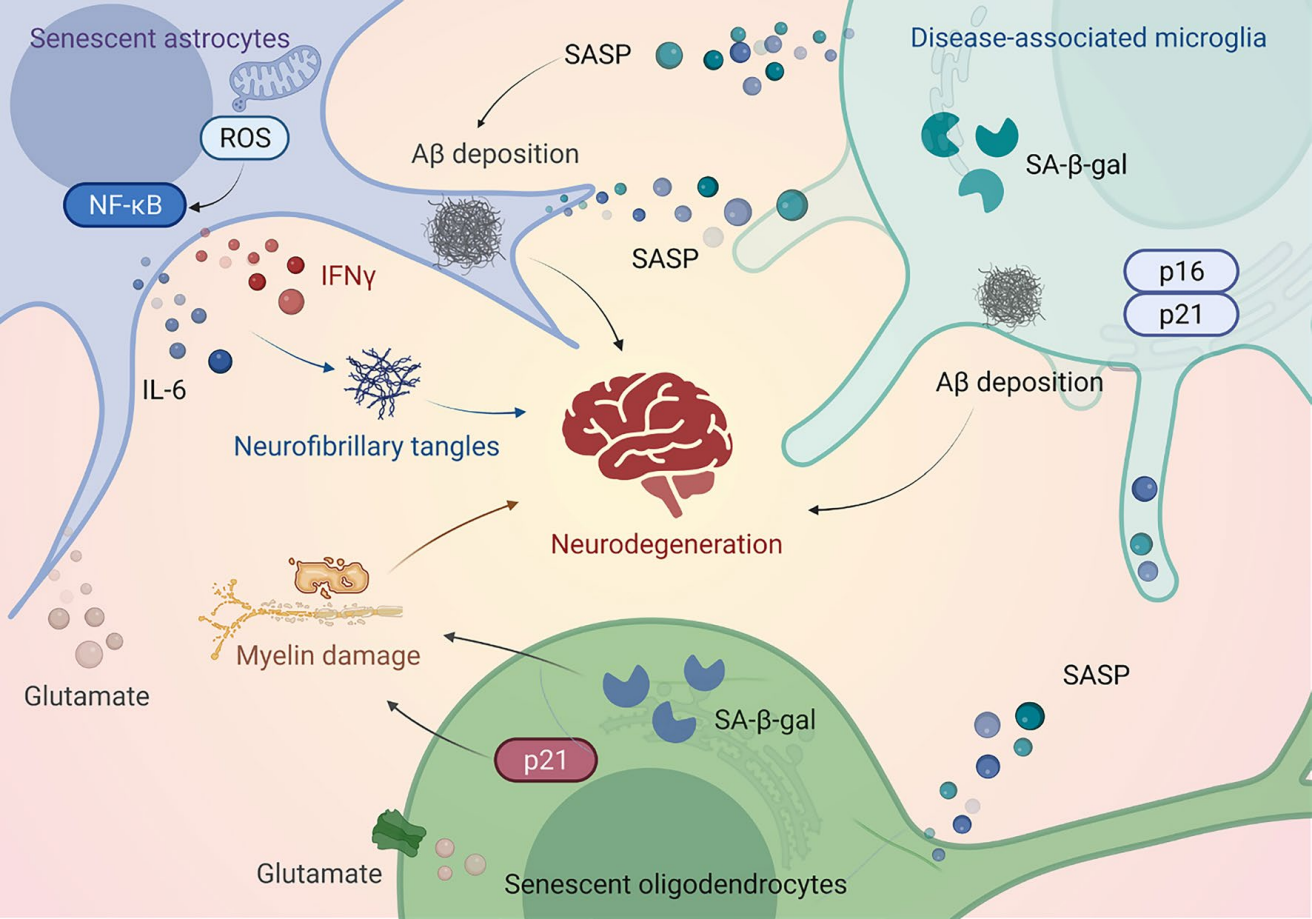
Brain-cell senescence contributes to neurodegeneration. Senescent astrocytes reveal dysfunctional mitochondria, elevating reactive oxygen species (ROS) levels to activate the nuclear factor kappa B (NF-κB) pathway and promote interleukin (IL)-6 and interferon-γ (IFNγ) secretions. This results in beta-amyloid (Aβ) accumulation and tau hyperphosphorylation, leading to neurofibrillary tangle formation. Furthermore, senescent astrocytes increase glutamate release and stimulate oligodendrocyte senescence, manifested as increased expression of the senescence markers, senescence-associated β-galactosidase (SA-β-gal), and p21. Oligodendrocyte senescence is responsible for myelin damage and contributes to neurodegeneration. Senescent astrocytes and oligodendrocytes secrete a sufficient SASP, which is detrimental to adjacent microglia. Senescent disease-associated microglia express elevated levels of SA-β-gal, p16, and p21, secrete SASP components, and contribute to Aβ pathology

The brain neuron count decreases with age (Zhou et al. 2022). SATB1 is a protein related to PD, loss of SATB1 causes p21-dependent cellular senescence in dopamine neurons, a contributing factor to PD pathology (Riessland et al. 2019). The deletion of growth differentiation factor 11 (GDF11) induces neuronal senescence by inducing p21 transcription (Wang et al. 2023). Selective knockout of GDF11 in excitatory neurons of mice induce neuronal senescence, overexcitation and synaptic input obstruction (Wang et al. 2023). Chronic alcohol abuse is a risk element for age-related dementia, and chronic alcohol metabolism lead to faulty neuronal DNA repair, promote nuclear accumulation of p21 and cyclin B, trigger cell cycle arrest and induce cell senescence (Sun et al. 2023). In addition, neuronal senescence correlates with AD neurodegeneration (Herdy et al. 2022). Neurons in patients with AD have senescence phenotypes, including increased p38/mitogen-activated protein kinase activity and transforming growth factor-β and IL-6 expression (Si et al. 2021). Neurons with NFTs have profiles consistent with cellular senescence in patients with AD (Musi et al. 2018). These involve downregulating cell death and upregulating pathways that promote survival and inflammation (Musi et al. 2018). Senescent cell-secreted SASP damages neuronal function and leads to neurodegeneration. Chronically elevated insulin causes dysregulation of p35 dynamics, culminating in the development of a senescence-like phenotype in neurons, with concomitant declines in brain function and cognitive performance (Chow et al. 2019). Furthermore, Aβ production may induce neuronal senescence (Hu et al. 2022). These beneficial effects have also been observed in neurologic disorders, and removing senescent-like neuronal cells improves chemotherapy-associated peripheral neuropathy in mice (Acklin et al. 2020). Furthermore, persistent microglial proliferation is a critical sign of AD (Hu et al. 2021). Microglial over-proliferation produces disease-associated microglia (DAM) in senescence, supporting early Aβ pathology in AD (Hu et al. 2021). Knockout of IL-1α, tumor necrosis factor-alpha (TNF-α), and C1q released from senescent microglia prolonged the survival time of SOD1G93A ALS mouse model (Guttenplan et al. 2020), indicating that targeting SASP improves disease phenotype in ALS mouse model (Granucci et al. 2019). Microglia with senescence markers emerged in the ALS rat model compared with normal rats, with a four-fold increase in p16-positive nuclei and a one to two-fold increase in lamin B1-negative nuclei, leading to motor neuron death. Moreover, pathologic remission occurs after drug clearance of senescent cells (Trias et al. 2019). These findings suggest that senescent cells mediate key pathogenesis in ALS. In addition, whole-body senescent cell removal from mice reduced microglial-and SASP-activation, alleviating age-related brain inflammation and cognitive impairment (Ogrodnik et al. 2021). Therefore, various brain cell types, including neurons, microglia, astrocytes, and neural stem cells, in a senescent state in NDs indicate that these senescent cells may be crucial in ND pathologic process (Fig. 1). Removing senescent cells attenuates neurodegeneration in murine AD and PD models, suggesting their contribution to neurodegeneration. Therefore, targeting senescent brain cells represents a novel therapeutic approach for NDs.

## Cellular senescence promotes Aβ accumulation in the AD brain

Recent evidence suggests that Aβ-driven cellular senescence is a complex cellular response mechanism in AD progression (Shang et al. 2020). In addition, senescent cells exacerbate Aβ pathology (Hu et al. 2021). Early responses to amyloid lesions include DAM phenotype emergence and microglial proliferation reactivation (Fuger et al. 2017). Microglial over-proliferation induces replicative senescence, leading to the DAM phenotype (Hu et al. 2021). This suggests that replicative senescence determines DAM emergence and contributes to Aβ pathology. In addition, Aβ plaques trigger OPC senescence observed in AD mouse models and the brains of patients with AD (Zhang et al. 2019a). Selective clearance of senescent cells from AD mice reduces neuroinflammation, attenuates Aβ load, and ameliorates cognitive deficits (Zhang et al. 2019a). Amyloid precursor protein (APP)/PS1 mouse models exhibited elevated brain Aβ load and memory damage. Moreover, various brain cells in AD mouse models expressed high levels of senescence markers, including p16, p21, and SA-β-gal (Hou et al. 2021; Jurk et al. 2012; Zhang et al. 2019c).

**Fig. 2:**
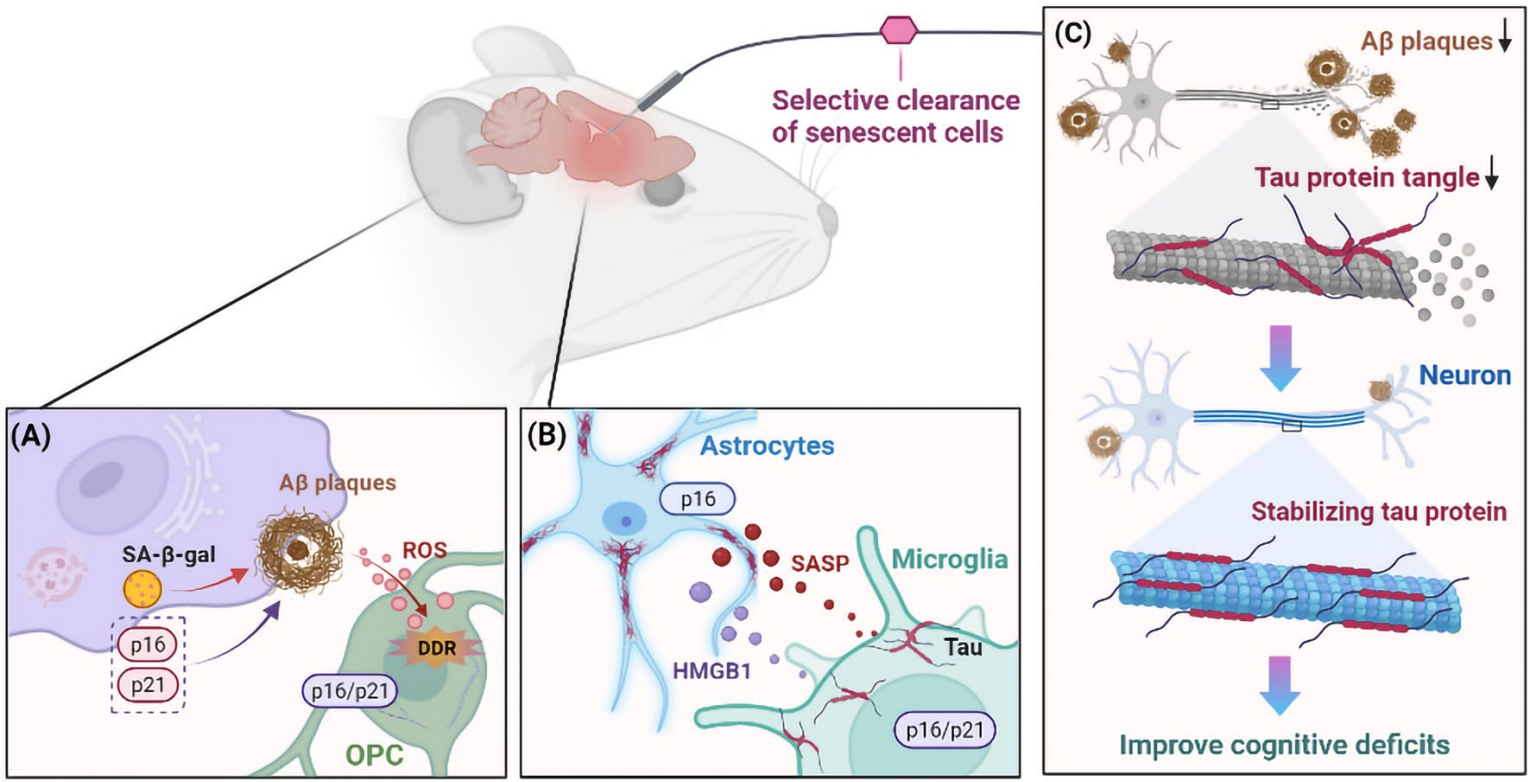
The role of cellular senescence in forming Aβ and tau proteins. **A** In the Alzheimer’s disease (AD) mouse model, senescent brain cells express elevated SA-β-gal, p16, and p21 levels. These phenotypes contribute to Aβ pathology, and Aβ plaques induce oligodendrocyte progenitor cellular senescence by increasing reactive oxygen species (ROS) production, resulting in the activation of the deoxyribonucleic acid (DNA) damage response (DDR) and p16 and p21 upregulation. **B** P16-positive senescent astrocytes and microglia accumulate in tau-dependent neurodegenerative diseases. Tau oligomers trigger cellular senescence by releasing SASP and HMGB1. **C** Selective clearance of senescent cells from AD mice attenuates Aβ load and prevents the deposition of tau-protein tangles, thereby improving cognitive deficits

In addition to these Aβ deposition mouse models, in vitro studies indicated that Aβ oligomers trigger neuronal or endothelial cellular senescence in culture media (Zhang et al. 2019a). Direct exposure of cultured OPCs to accumulated Aβ induces cellular senescence (Zhang et al. 2019a). Vascular endothelial growth factor receptor-1 modulates Aβ1-42 oligomer-induced senescence in brain endothelial cells (Angom et al. 2019). Aβ promotes neuronal cellular senescence by increasing ROS production (Zhang et al. 2017). Similarly, adult mouse hippocampal neural stems or progenitor cells treated with Aβ1-42 oligomers increase p16 and SA-β-gal (He et al. 2013). Moreover, treating rat astrocytes with Aβ increases IL-1β, a neuroinflammatory cytokine that increases during cellular senescence (Shang et al. 2020), indicating that Aβ promotes astrocyte senescence. These results suggest that cellular senescence plays a critical role in Aβ-mediated neuropathology and memory deficits (Fig. 2).

## Cellular senescence plays a crucial role in tau-mediated disease

Cellular senescence affects the onset and progression of tau-mediated disease (Mendelsohn and Larrick 2018). Dysfunctional glial cells directly contribute to neuronal tau pathology (Taddei et al. 2022; Vogels et al. 2019). In a tau-dependent ND mouse model, p16-positive senescent microglia and astrocyte accumulation were observed (Bussian et al. 2018). p19 was an important contributor to human brain senescence eigengene, p19-expressing neurons are highly expressed in AD brain tissues, compared with p19-negative neurons, the nuclear volume and lipofuscin are significantly increased, and overlap with tau-containing NFTs (Dehkordi et al. 2021). Eliminating these senescent cells protects cognitive function by preventing NFT deposition and cortical and hippocampal neuron degeneration (Bussian et al. 2018). Microglia can phagocytose, degrade, and remove extracellular tau proteins. Microglia senescence leads to immune dysfunction, reducing the efficiency of extracellular tau-protein scavenging, increasing tau-protein phosphorylation, and promoting tau pathology spread (Vogels et al. 2019). Removing these senescent glial cells in a neuronal tauopathy mouse model results in reduced tau pathology and enhanced cognition (Bussian et al. 2018).

Tau oligomers trigger cellular senescence via high mobility group box 1 (HMGB1) release and inflammatory SASP, contributing to neuropathology in AD and frontotemporal dementia (Gaikwad et al. 2021). Inhibiting p38/mitogen-activated protein kinase and NF-κB, the basic SASP pathways, prevents tau oligomer-induced senescence, alleviates neuroinflammation, and improves cognitive functions (Gaikwad et al. 2021). Elevated brain iron and iron dyshomeostasis are associated with AD pathology and cognitive decline, resulting in neurodegeneration by accelerating ferroptosis (Bao et al. 2021; Masaldan et al. 2019). Interactions between tau and iron are involved in forming NFTs (Wan et al. 2019). Iron overload can generate ROS, predominantly hydroxyl radicals, via the Fenton reaction, leading to oligomeric tau formation and hyperphosphorylation (Wan et al. 2019). After phagocytosis of neurons containing tau aggregates, microglia exhibit senescence-like phenotypes, such as SA-β-gal activity, secretion of matrix metalloproteinase-3, and release of tau seeds (Brelstaff et al. 2021). Therefore, cellular senescence is a key intermediate in tau-mediated neurodegeneration (Fig. 2). Cellular senescence may contribute to some pathologic features, such as elevated levels of pro-inflammatory molecules in nerve cells and synaptic dysfunction, associated with tau aggregation and neuronal degeneration (Derry et al. 2020).

## Telomere damage-induced cellular senescence mediates NDs

Telomeres found at the ends of mammalian chromosomes are shortened during cell replication, resulting in replicative senescence (Carey et al. 2022; Flynn and Heaphy 2019). Telomere attrition in the brain can lead to senescence and neuronal death, resulting in cognitive decline (Lv et al. 2019a). Telomere shortening is associated with rapid cognitive decline and progression to dementia in patients with mild cognitive impairment (MCI) (Koh et al. 2020). Shorter leukocyte telomere length increases MCI/AD risk (Ma et al. 2019). Serum folate concentration positively correlates with leukocyte telomere length, and folate deficiency may affect neuronal telomere dynamics, leading to cognitive decline (Ma et al. 2019). Patients with AD have shorter telomeres than age-matched healthy controls (Madrid et al. 2020). Mendelian randomization analysis revealed that a longer leukocyte telomere is associated with lower AD risk (Guo and Yu 2019; Madrid et al. 2020). Telomere shortening rates are higher in patients with AD than in those with MCI and healthy individuals, as only those with AD show a noticeable telomere shortening of 60 bp annually (Lee et al. 2021). APP/PS1 mice showed substantial telomere shortening in DAM compared with homeostatic microglia (Hu et al. 2021). In addition, a negative relationship was observed between age and telomere length (Lee et al. 2021). The average rate of leukocyte telomere shortening reported in previous studies ranged from 23 to 47 bp annually (Chen et al. 2011). The average telomere shortening increases by approximately 37 bp with age yearly in Koreans (Lee et al. 2021). Mice with experimental nerve damage can display long-term male-specific neuropathic pain, with telomere length reduction and p53‑mediated cellular senescence in the spinal cord, after repeated injection of p53-specific senolytic peptide administration, the pain response of male mice was reversed (Muralidharan et al. 2022).

Therefore, restoring telomere length is pivotal for cell proliferation and DNA stability. Telomerase, comprising telomerase reverse transcriptase (TERT) and the telomerase RNA component (TERC), may protect against tau pathology in patients with AD (Spilsbury et al. 2015). Telomerase gene therapy delivered to the brains of telomerase-deficient mice improves several neurodegeneration phenotypes (Whittemore et al. 2019). In contrast, TERT deficiency induces APP and reduces brain-derived neurotrophic factors in the mouse brain (Shim et al. 2021). TERT activation alleviates amyloid pathology (Shim et al. 2021). Neuronal TERT expression improves dendritic spine formation and cognitive function in aging AD mouse models (Shim et al. 2021). TERT triggers the β-catenin/TCF7 complex, upregulates gene networks governing synaptic signaling and learning, and offers neuronal protection amid toxic Aβ accumulation in human and mouse AD models (Shim et al. 2021).

**Fig. 3:**
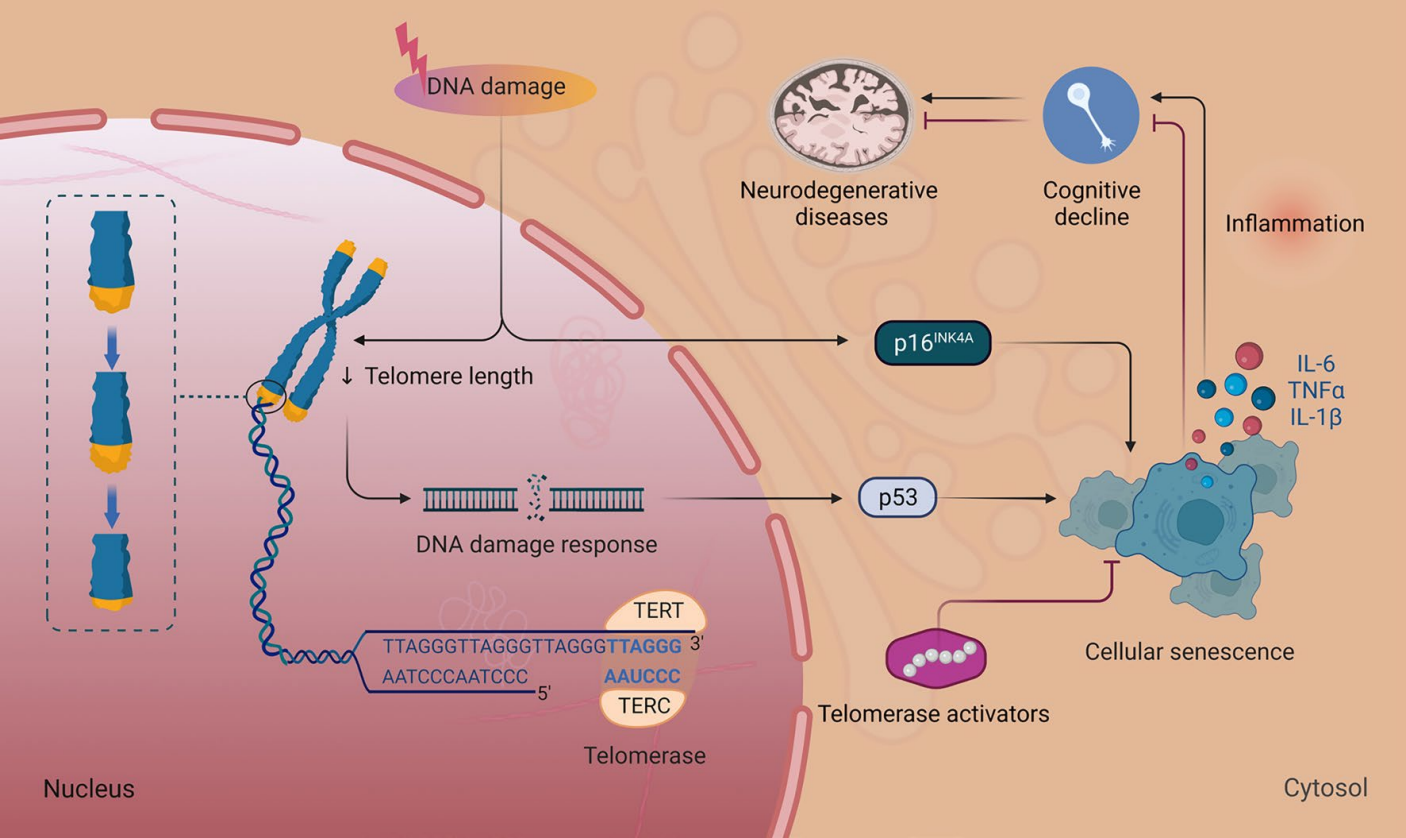
Telomere damage-induced senescence promotes neurodegenerative diseases. DNA damage leads to the gradual shortening of telomeres, one or a few DNA damage response (DDR) signaling telomeres, the ends of chromosomes, and signals finally converge on p53 and p16 activation, causing cell cycle arrest. Eventually, sustained DDR activation induces cellular senescence. Telomere dysfunction causes cellular senescence, which stimulates IL-6, TNF-α, and IL-1β secretions. The intertwined process of senescence and inflammation is correlated with cognitive decline and leads to neurodegenerative diseases. Telomerase comprises telomerase reverse transcriptase (TERT) and telomerase ribonucleic acid components (TERC). Forced expression of telomerase activators prevents cellular senescence, showing substantial neuroprotective effects, which rescues degenerative diseases with critically shortened telomeres

Furthermore, ROS-induced telomere damage may trigger telomere dysfunction-induced focal generation and senescence (Chakravarti et al. 2021). Aging incorporates inflammatory signals from genomically damaged or senescent cells. Telomere dysfunction can activate and sustain inflammation, trigger cellular senescence, and spur the formation and secretion of IL-6 and TNF-α, as well as the additional inflammatory factors, IL-1β and PAI-1 (Guerrero et al. 2021). The intertwined senescence and inflammation processes correlate with the telomere-aging connection (Fig. 3) (Chakravarti et al. 2021). Telomere shortening causes senescence, fibrosis, and inflammation, leading to aging and other degenerative and inflammatory diseases (such as AD, PD, multiple sclerosis, and ALS). Telomerase activators and senolytics mitigate these lesions, reducing aging and age-related diseases. As an illustration, folic-acid supplementation delays neurodegeneration in senescence-accelerated mice (Lv et al. 2019b) owing to mitigated telomere depletion in the hippocampus and cortex (Li et al. 2022). In addition, folic-acid supplementation decreases apoptosis and telomere depletion in primary astrocyte cultures, elevating telomerase activity (Li et al. 2022).

## Targeting cellular senescence to diagnose and treat NDs

Based on the aforementioned mechanisms, targeting cellular senescence is a promising approach for alleviating and treating NDs. Some agents, such as senotherapeutics, which show promising therapeutic effects on NDs by regulating cell senescence processes, are gaining attention and yielding positive results in animal models and clinical practice (Gonzales et al. 2022; Hou et al. 2022). Recent studies have revealed the effectiveness of interventions, such as young blood and CSF exchange, in mitigating ND progression (Iram et al. 2022; Zhang et al. 2023a). Furthermore, gut-microbiota transplantation has provided further insights into ND treatment (Boehme et al. 2021; Westfall et al. 2017). Therefore, targeting senescence and specific cell demise may be a novel approach for treating NDs.

Senotherapeutics encompass two strategies: senolytics, which selectively remove senescent cells and induce apoptosis to improve tissue function, and senomorphics, which regulate senescent cells to neutralize harmful effects by suppressing the expression levels of SASP or specific SASP mediators (Wissler Gerdes et al. 2020). A number of agents from the two strategies have been shown to be successful in slowing the progression of a number of age-related diseases, such as NDs (Table 1). A typical senolytic therapy used in NDs is dasatinib plus quercetin (D + Q) (Gonzales et al. 2022; Hickson et al. 2019). D + Q induces apoptosis in senescent OPCs and attenuates IL-1β and TNF-α expression levels in mice (Gonzales et al. 2022; Zhang et al. 2019b). In the first phase I clinical trial (NCT04063124), D + Q oral administration decreased SA-β-gal activity and the expression levels of p16^INK4A^, p21^CIP1^, and SASP factors, including IL-1α, IL-6, MMPs-9, and MMPs-912 (Gonzales et al. 2023; Hickson et al. 2019). A larger phase II clinical trial (NCT04685590) used an intermittent dosing strategy to establish the safety and efficacy of 12-week D + Q treatment in older adults with early-stage AD (Gonzales et al. 2022). AP20187, a compound targeting cellular senescence and anti-inflammation, induces apoptosis in high p16^INK4A^-expressing cells, reducing senescent microglia and improving cognitive function in aged mice (Alsuraih et al. 2021; Ogrodnik et al. 2021). Serum/glucocorticoid related kinase 1 (SGK1) is upregulated in the brains of patients with ND, and its inhibition may be a useful strategy for treating PD and other NDs with glial cell-mediated neuroinflammation (Jang et al. 2022; Kwon et al. 2021). The SGK1 inhibitor, GSK-650394, enhances glial cell activity by scavenging glutamate toxicity, preventing cellular senescence, and mitigating mitochondrial damage (Kwon et al. 2021). Interestingly, senescence may be a cause of brain dysfunction after brain injury in a sex-dependent manner (Schwab et al. 2022). Treatment of mice with ABT263 could only significantly reduce senescence markers of male mice and significantly improve the Morris water maze performance, but had no therapeutic effect on female (Schwab et al. 2022). The senescent cell-secreted SASP is a predisposing factor for various NDs, highlighting senomorphics (Birch and Gil 2020). Rapamycin, an inhibitor of the mammalian target of rapamycin (mTOR), improves cerebrovascular and cognitive function in AD mice (Van Skike et al. 2018). Ginsenosides improve brain function, especially in AP and PD (Chen et al. 2020; Zhang et al. 2022). Ginsenoside F1 reduces IL-6 and IL-8 secretions via astrocytes (Hou et al. 2018). Moreover, ginsenoside F1-enhanced mixture, SGB121, reduces IL-6, IL-1β, and TNF-α secretions, attenuates inflammatory responses, and abates excessively phosphorylated tau protein in the cortex (Hou et al. 2022). TNF-α increases Aβ and tau production and induces cognitive dysfunction (Patlola et al. 2023). Adalimumab inhibits TNF-α activity and reduces inflammatory responses (Prattichizzo et al. 2016). Adalimumab treatment reduces β-secretase-1 (BACE1) expression and Aβ_1-40_ plaques, enhancing memory in Aβ_1-40_-injected mice (Park et al. 2019). Overall, SASPs vary, and many drugs regulate SASPs by regulating epigenetic modifications in transcriptional and post-transcriptional pathways (Hernandez-Segura et al. 2018). Senomorphics primarily target the SASP by inhibiting the NF-κB transcription factor or using mTOR inhibitors (Lagoumtzi and Chondrogianni 2021). Ongoing studies are investigating the mechanisms of action of these drugs, requiring further investigation and validation to establish their efficacy and safety for clinical trials (Kakoty et al. 2023). Therefore, the use of senotherapeutics is a promising strategy (Lagoumtzi and Chondrogianni 2021).

**Table 1:** Potential drugs against cellular senescence to NDs therapy

Substance		Model	Targets	Neurophysiological behavioral outcomes	References
Senolytics	Dasatinib plus quercetin (D + Q)	Adults aged 65 years and older with diagnosed early-stage AD	Phase II, determine BBB penetrance of 12-week (D + Q) treatment. (NCT04063124; NCT04685590)	Intermittent dosing strategy being utilized, determine safety and efficacy in older adults with MCI or early-stage AD	Gonzales et al. (2022)
		AD transgenic mouse	Targeting cellular senescence to treat tau‐associated neurodegeneration mice in late life	↓ Neurofibrillary tangle burden, ventricular enlargement, and neurodegeneration in 23-month-old tau transgenic mice	Musi et al. (2018)
		APP/PS1 transgenic mice	Selectively removed senescent cells from the plaque environment	↓ Neuroinflammation ↓ Aβ load ↓ Cognitive deficits	Zhang et al. (2019b)
	AP20187	*INK-ATTAC* transgenic mouse model	Apoptosis of cells with high expression of p16^INK4A^	↓ Senescent microglial population ↓ Microglial activation and expression of SASP factors ↑ Cognitive function in aged mice	Ogrodnik et al. (2021)
	GSK-650394	PD mouse model, in vitro αsynucleinopathy models	Serum/glucocorticoid related kinase 1 (SGK1) inhibitor, corrects the pro-inflammatory properties of glia	↓ NFκB-, NLRP3-inflammasome-, and CGAS-STING-mediated inflammatory pathways in glia ↓ Glial cell senescence and mitochondrial damage ↓ Pathologic α-synuclein aggregation ↓ PD-associated behavioral deficits in multiple in vitro and in vivo PD models	Kwon et al. (2021)
	ABT263	*PS19* mouse model	Administered orally at a dose of 50 mg per kg body weight for 5 consecutive days of treatment in mouse of tau-dependent neurodegenerative disease	↓ Cell-cycle regulators *p16*^*INK4A*^*, p19*^*Arf*^*, p21*^*Cip1/Waf1*^ ↓ Pro-inflammatory genes *Pai1, IL-6* and *IL-1β* ↓ Tau phosphorylation	Bussian et al. (2018)
		Adult (8–10 week old) female and male C57BL/6 mice	Induced mild closed-skull injuries or sham procedures	↑ Male mice performance in the Morris water maze ↓ p21 ↔ Senescence markers in female mice	Schwab et al. (2022)
Senomorphic	Rapamycin	AD mouse models	Inhibition of the mTOR pathway	↑ Neuroprotective effect ↑ Cerebrovascular and cognitive function	Van Skike et al. (2018)
	Ginsenoside F1	Astrocytic CRT and U373-MG cells, human primary astrocytes and SH-SY5Y cells, C57BL/6J mice	Suppressing p38MAPK-dependent NF-κB activity	↓ Secretion of IL-6 and IL-8 from astrocytes ↓ IL-6, IL-1β and TNF-α ↓ Phosphorylated tau in mouse brain ↔ Aβ levels in mouse brain	Hou et al. (2018); (Hou et al. 2022)
	Adalimumab	Male mice (30–35 g; 7 weeks old)	Directly bind to TNF-α and block receptor binding, inhibit TNF-α activity	↓ β-secretase-1 (BACE1) protein ↓ Aβ_1-40_ plaques ↓Neuronal damage and neuroinflammation in Aβ_1-40_-injected mice	Park et al. (2019)

↑ increased/improved; ↓ reduced/decreased; ↔ unchanged

Blood-exchange therapy may reverse aging in ND treatment. Components in the blood of young individuals contribute to vitality, and isolated factors may be used as therapies to extend health and lifespan (Sha et al. 2019; Zhang et al. 2023a). Heterochronic parabiosis (HPB) is a state in which live animals, one young and one old, are connected to share a circulatory system (Poganik et al. 2023). A study demonstrated that after connecting young and old mice with HPB for 3 months, the older mice slowed the aging process at the cellular level and increased their lifespan by 10%, with this rejuvenating effect continuing after 2 months (Zhang et al. 2023a). HPB rejuvenates certain mouse tissues; however, its overall long-term health effects remain unknown. Human umbilical cord plasma treatment rejuvenates the hippocampus and improves cognitive function in aged mice (Castellano et al. 2017). Mice and humans experience a substantial decline in blood extracellular nicotinamide phosphoribosyltransferase (eNAMPT) levels with age (Park et al. 2023). Supplementing aged mice with eNAMPT from younger mice promotes whole-body nicotinamide adenine dinucleotide (NAD^+^) biosynthesis, counteracts senescence, and improves memory and cognitive function (Yoshida et al. 2019). However, the effects of exchanging young plasma for patients with AD remain unknown (Yoshida et al. 2019; Zhang et al. 2023a). A randomized clinical trial of plasma infusion in young adults to improve AD symptoms (NCT02256306) suggested that young fresh-frozen plasma treatment is safe, well-tolerated, and feasible (Sha et al. 2019). Blood–brain interactions are complex multidisciplinary issues. Therefore, translating these scientific findings into clinical practice requires a deeper understanding of the molecular targets, safety, tolerability, and ethical implications to fully appreciate the complications and effectiveness of this therapeutic strategy.

CSF replacement therapy may represent a potential treatment for NDs. To modulate brain function, systemic factors originating from sources (such as HPB or plasma transfer) cross barriers or conduits to reach brain cellular targets, with subsequent clearance from the parenchyma or CSF into the periphery (Cruz Hernandez et al. 2019; Pluvinage and Wyss-Coray 2020). Directly infusing young CSF into the aging brain can improve memory function in aged mice (Iram et al. 2022). Young CSF promotes OPC proliferation and differentiation (Iram et al. 2022). Fibroblast growth factor 17 (Fgf17) infusion, a potential serum response factor (SRF) activator in the CSF, induced OPC proliferation and long-term memory consolidation in aged mice, whereas Fgf17 blockade impaired cognitive performance in young mice (Iram et al. 2022). Further studies are needed to explore the effects of SRF on other cell types. Ongoing research aimed at enriching our understanding of factors present in young CSF that can prevent or rescue cognitive decline holds promise as a novel therapeutic strategy.

Gut flora transplantation may alleviate ND symptoms. Gut microbiota can have deleterious effects in neurodegenerative conditions, causing localized immunity, affecting brain aging, and increasing the risk of developing NDs (Pluvinage and Wyss-Coray 2020). The gastrointestinal tract communicates with the CNS via the gut–brain axis (Westfall et al. 2017). Transplantation of fecal microbiota from young (3–4 months) donor mice into aged (19–20 months) recipient mice attenuated age-related selective cognitive behavioral deficits in the aged host (Boehme et al. 2021). In addition, hippocampal neurogenesis is a critical process for learning and memory, and the surviving newborn hippocampal neurons are reduced in aged mice; however, fecal microbiota transplantation cannot rescue this phenomenon (Boehme et al. 2021). The symbiotic microorganisms in the gastrointestinal tract are beneficial and essential for health; however, the potential enterotoxicity of these microorganisms cannot be ignored. For example, lipopolysaccharides (LPSs) are microbial-derived glycolipids that are pro-inflammatory neurotoxins (Peng et al. 2021). LPSs and other endotoxins produced by microorganisms can cross the biophysiological barrier of the gastrointestinal tract, enter the systemic circulation, and cross the blood–brain barrier (Peng et al. 2021). Further evidence suggests that LPSs upregulate the pro-inflammatory transcription factor complex NF-κB (p50/p65) and subsequently generate a series of NF-κB-sensitive microRNA upregulations (Zhao et al. 2022). Understanding the molecular genetic signaling of the gastrointestinal microbiome in healthy aging patients and those with AD holds potential for developing novel diagnostic strategies and monitoring treatment efficacy (Zhao et al. 2022). These studies suggest a link between aging and the gut microbiota. Thus, the microbiome may be a suitable therapeutic target for promoting healthy aging.

Current treatments for NDs are not ideal; however, research on treatments targeting cellular senescence continues to advance, showing promising trends and prospects. Overall, these results suggest that cellular senescence may be clinically applicable for patients with NDs (Gonzales et al. 2022). However, currently developed medicines that target cell senescent to diagnose and treat NDs are limited. The application of new intervention strategies to slow down brain aging is gradually emerging. Further crossover studies and larger clinical trials are required to validate the efficacy and safety of these approaches.

## Major challenges for future experiments

Senescent cells in vivo are heterogeneous, necessitating future technological advancements for accurate identification and characterization. These advancements include single-cell/nucleus RNA-sequencing methods and additional single-cell resolution techniques (Cohn et al. 2022; Xu et al. 2022). Purposefully research drugs targeting different types of neuronal cell senescence still require extensive trial screening, such as rosemarinic acid was found to reduces microglial cell senescence for control of neuropathy symptoms (Borgonetti and Galeotti 2022). Data analysis can benefit from traditional and net meta-analyses applied to create an accessible and comprehensive database and systematically evaluate and summarize the relationship between cellular senescence and NDs, offering a novel strategy for ND treatment (Zhao et al. 2019). Currently, elucidating the cellular senescence mechanism in NDs is primarily based on in vitro cellular experiments and animal models. However, monolayer cell structure in traditional two-dimensional culture techniques does not accurately reproduce physiologic or pathologic processes in animals (Vijaya et al. 2023). Constructing a three-dimensional (3D) ND model offers improved simulation of complex neural networks and inflammatory responses of the brain. However, the main challenge of 3D neural tissue culture is the lack of vascularization of the 3D cell model (Osaki et al. 2018), which is not conducive to constructing a disease model for advanced patients. Moreover, late-stage drug experiments require animal model support.

Animal models are crucial in studying human senescence mechanisms and drug development. Compared with humans, using rodent models to simulate progressive diseases of the human nervous system has limitations, such as individual differences between mice, shorter lifespans, rapid breeding cycles, and differences in body structure and genetics (Eaton and Wishart 2017). Several therapeutic approaches have demonstrated efficacy in rodent models; however, most have failed in human clinical trials. Owing to the complex ND pathogenesis, they may be a consequence of complex environmental and genetic factors. However, using rodents as models of neurologic diseases has several limitations. Large animal models often better mimic human neurologic disorders than rodent models in terms of phenotype and pathology. Accordingly, when investigating possible ND therapies, utilizing human cell-based and large animal models is crucial (Eaton and Wishart 2017). Non-human primate models with higher genetic homology can be used (Hasselmann et al. 2019). However, from an economic point of view, creating large animal models is expensive, and general laboratories do not have the conditions to create such models.

## Conclusions and perspectives

**Fig. 4:**
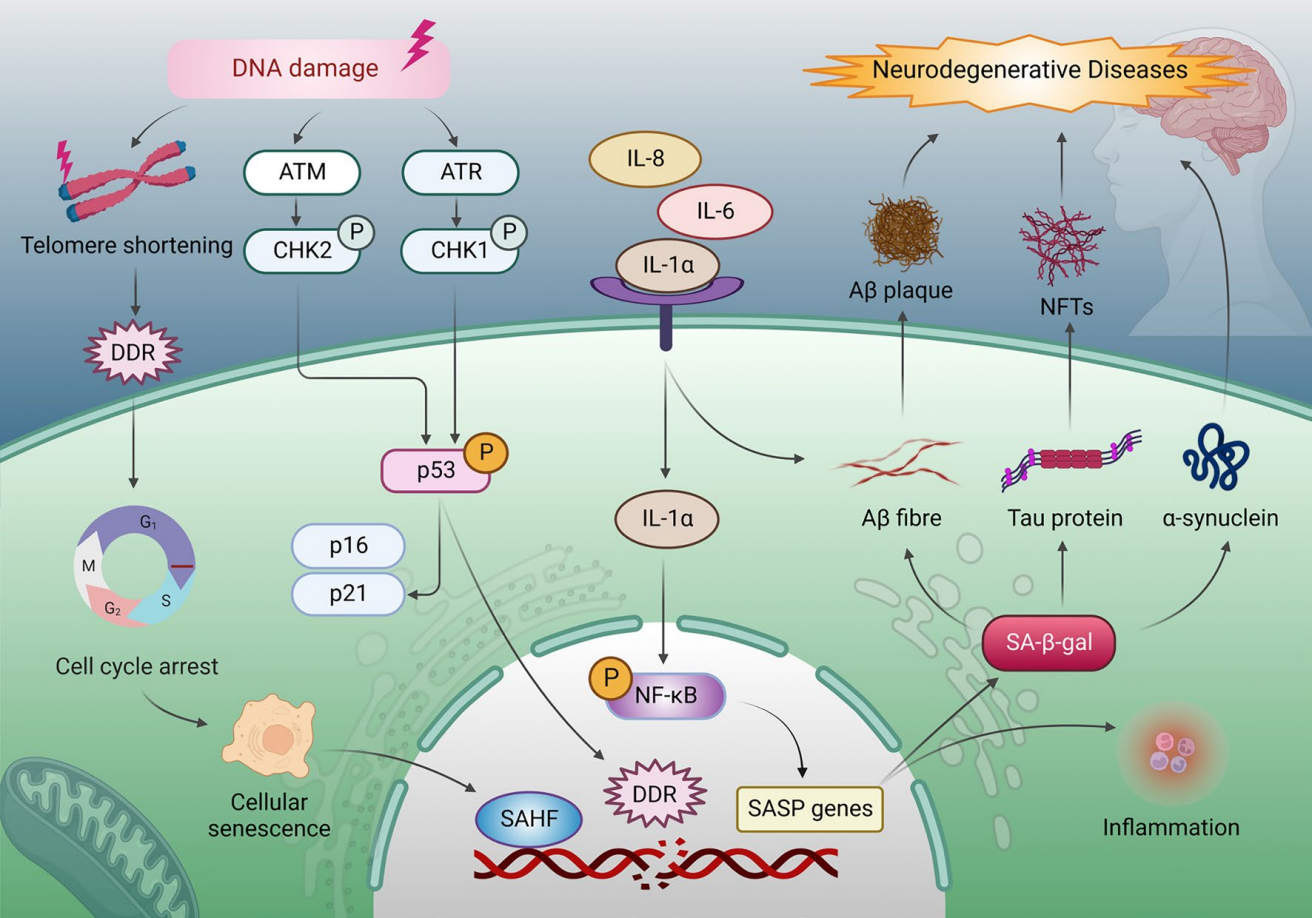
Cellular senescence drives neurodegenerative diseases. DNA damage accumulates at the telomere, where telomere shortening activates the DNA damage response (DDR), triggering cellular senescence and generating senescence-associated heterochromatin foci. DNA damage is related to the apical kinases, ATM and ATR, and the downstream kinases, CHK2 and CHK1. Subsequently, signals converge upon p53 activation, which causes cell cycle arrest and governs p21 and p16 expression. Prolonged DDR activation triggers senescence. Pro-inflammatory IL-8 and IL-6, mediated in an IL-1-dependent manner, induce SASP genes by enhancing NF-κB activity, regulating SASP drives inflammation and SA-β-gal. Accumulated inflammatory cytokines and SA-β-gal aggravate Aβ fibers, tau protein, and α-synuclein, promote Aβ deposition and NFT formation, finally inducing neurodegenerative disease

The increase in senescent cells in various NDs underscores their importance in the pathophysiology of these diseases (Fig. 4). In addition, cellular senescence alters proteostasis in certain diseases, leading to abnormal protein aggregates or misfolded proteins. Telomere dysfunction, DNA damage, oxidative stress, and neuroinflammation contribute to ND pathogenesis. Pathologically induced and age-related senescence are possible origins of senescent cells in the brains of patients with AD. Eliminating senescent cells or some ND pathologic markers can mutually influence outcomes.

Cellular senescence is an intriguing phenomenon in NDs, making senotherapy a compelling treatment option. Senolytic therapy attenuates senescence in Aβ-associated OPC and cognitive dysfunction in an AD model (Zhang et al. 2019a). Targeting senescent cells offers a therapeutic approach for NDs. However, developing clinically effective drugs remains challenging and requires multimodal targeting. Detailed studies on the mechanisms of action of these drugs are ongoing and have not been fully elucidated. Caution and reliance on additional scientific data and clinical findings are required when using and evaluating these compounds. Combining high-resolution molecular techniques such as small conditional RNA-sequencing, unbiased proteomics, and cell-type-specific gene manipulation with holistic physiologic system analysis and cross-tissue communication may improve our understanding of the complex interplay between cellular senescence and NDs (Pluvinage and Wyss-Coray 2020). The limitations of this clinical blood exchange study include its small sample size, short duration, and restricted effectiveness analysis. Therefore, these findings warrant further exploration in larger, double-blind, placebo-controlled clinical trials, including an analysis of dosage, pharmacokinetics, and pharmacodynamics (Sha et al. 2019). With the continuous development and innovation of science and technology, a more effective therapeutic approach against cellular senescence will emerge to prevent and treat NDs.

The accumulation of various senescent nerve cells triggers chronic inflammation, and SASP secretion promotes tau-protein accumulation and Aβ deposition, contributing to the development of NDs, such as AD. However, several questions remain unanswered, such as the unclear properties of the senescent secretome in the brain. In addition, further investigation is needed to understand the effect of modulating SASP on improving neurodegeneration (Keshavarz et al. 2023). The role of cellular senescence in AD pathogenesis remains unclear; however, eliminating senescent cells in the nervous system alleviates AD pathology and improves cognitive function in AD mice. This suggests that cellular senescence may be a reliable therapeutic target for AD. Accurately identifying senescent cells in vitro and in vivo is the initial step in promoting an understanding of the diverse effects of cellular senescence types on AD pathogenesis.

Oligodendrocytes primarily produce the myelin sheath, the membrane that surrounds the axons of nerve cells. Aging myelin sheaths release myelin pieces, which are subsequently eliminated by microglia (Safaiyan et al. 2016). Accumulated myelin fragmentation causes lipofuscin-like lysosomal inclusions in microglia, resulting in microglial senescence and immune dysfunction with aging (Safaiyan et al. 2016). The nervous system is sensitive to hypoxia (Liu et al. 2021). Hypoxia damages the myelin sheath, leading to neuronal loss (Wang et al. 2018). In addition, hypoxia and the activation of hypoxia-inducible factors trigger signaling cascades that regulate the secretion of immunosuppressive cytokines and growth factors for immune escape (Wu et al. 2022). Therefore, elucidating the mechanisms underlying cellular senescence in a hypoxic microenvironment is necessary.

## Data Availability

Not applicable.
